# Downward gazing behavior after stroke can enhance postural control even in the absence of visual input

**DOI:** 10.3389/fneur.2025.1593221

**Published:** 2025-05-14

**Authors:** Yogev Koren, Simona Bar-Haim, Noy Goldhamer, Lior Shmuelof

**Affiliations:** ^1^Department of Cognitive and Brain Sciences, Ben-Gurion University of the Negev, Be’er-Sheva, Israel; ^2^Department of Physical-Therapy, Ben-Gurion University of the Negev, Be’er-Sheva, Israel; ^3^The Lillian and David E. Feldman Research Center for Rehabilitation Sciences, Adi Negev-Nahalat Eran Medical Center, Ofakim, Israel

**Keywords:** postural control, stroke, vision, downward gazing, sensorimotor control, standing sway, gaze angle

## Abstract

**Background:**

Recent reports have revealed that downward gazing, a common behavior among persons with stroke, enhances postural control. The mechanism underlying this phenomenon is currently unknown. In this study, we attempt to provide evidence to support the hypothesis that this effect is primarily derived from altered retinal input caused by gazing down. We also hypothesized that the effect of downward gazing on sway will be more pronounced in subjects with impaired balance control following stroke.

**Methods:**

We quantified standing postural sway of 20 healthy participants and 20 persons with stroke who were instructed to stand as still as possible under different conditions: while gazing forward and gazing down, with their eyes open and eyes closed.

**Results:**

Both the horizontal gaze angle and the lack of visual input had a negative effect on participants’ ability to attenuate their body sway. Yet, the effect of gaze angle was constant regardless of the presence or absence of visual input. Also, people with stroke were more sensitive to the effect of gaze angle.

**Discussion:**

The results of this study indicate that downward gazing enhances postural control even in the absence of visual input and do not support our main hypothesis. Nonetheless, the effect of downward gazing on postural control was greater in unstable people (persons with stroke) than that observed in healthy adults, supporting our secondary hypothesis, which might explain less stable individuals’ tendency to gaze down while walking.

## 1 Introduction

It is generally accepted that somatosensory, vestibular and visual information are integrated and used to control posture (1). That is, these sensory modalities provide information about the body’s position and motion that is used to generate corrective responses to gravitational and other internal and external forces acting on the body. Given that the visual and vestibular organs are in the head, and that sensory information from the muscles controlling eyes and neck have been implicated in postural control (2), any change in gaze position can affect the signals provided by these modalities, which in turn, can affect postural control.

Although during daily life activities humans often change their gaze position, gaze behavior during walking is mostly studied in the context of anticipatory stepping control (3). Downward gazing (DWG) while walking is a common clinical observation among persons with stroke (PwS) (4), and in other unstable walkers, but very few investigations were conducted to determine how this gaze behavior affects postural control (2, 4–7). Moreover, observations from these investigations were inconsistent and sometimes conflicting, with various mechanisms proposed to underly the observed effects (5, 6).

Recently, Koren et al. (8) reported that DWG enhanced postural steadiness of standing and walking younger adults. These authors also found a similar effect with older adults and PwS (9), two populations that excessively rely on visual input and are more likely to gaze down while walking (10, 11). Based on previous literature and deductive reasoning, they speculated that DWG enhances postural control primarily through its effect on the visual input [for a comprehensive explanation see (8)]. In this investigation we attempted to provide evidence to support this speculation. To do so, we tested whether the effect of DWG on postural sway with visual input (eyes open) was different from the effect observed without visual input (eyes closed). Specifically, we hypothesized that the effect of DWG on postural sway with eyes open would be greater than the effect with eyes closed (if such an effect is even observed). In other words, we expected to find a Vision by (gaze) Angle interaction, a prediction that previous reports had not tested directly. We also tested whether the effect of DWG is more pronounced in PwS, as PwS are less stable and tend to rely excessively on visual input. For this purpose, a significant Group by (gaze) Angle interaction was considered as supportive.

## 2 Methods

### 2.1 Participants

Twenty healthy adults and 20 PwS participated in this study. Participants were recruited at the Adi-Negev Rehabilitation Centre from among the patients (in-and outpatients), staff, and visitors (i.e., a convenience sample). All participants provided written consent prior to testing. Participants were men and women between 18 and 85 years old, able to stand with their eyes closed for 30s, able to provide consent, and able to follow simple instructions. Exclusion criteria included: (1) history of a major orthopedic condition (such as total knee replacement), or present acute orthopedic symptoms (such as severe pain due to osteoarthritis). (2) Any neurological (other than stroke) or degenerative condition. (3) Any other conditions that can affect postural control, such as vertigo, severe visual impairment, etc. Participants with common age-related conditions, such as controlled type 2 diabetes, hypertension, etc., were allowed to participate. The study complies with standards of the Declaration of Helsinki including approval of an institutional review board (regional ethical review board at Sheba Medical Center, Israel, approval number 6218-19-SMC).

### 2.2 Procedure

Participants were instructed to stand barefoot, as still as possible, in a standardized wide-base stance, i.e., their heels 6 cm apart with the feet externally rotated (10°), and their hands loosely hanging to the sides of their bodies (12). Their postural sway was measured while gazing forward or downward, with their eyes closed or open, for 30s in each trial. This can be visualized as a 2 × 2 conditions matrix (2 visual conditions and 2 angle conditions). For the forward condition, participants gazed at a target placed at eye level, located 4 meters ahead, and for the downward condition the target was placed on the standing surface, 2 meters ahead. The gaze targets were colored circles, 20 cm in diameter, made of laminated paperboard. In the eyes-open condition, participants were instructed to gaze at the designated target constantly throughout the trial. In the eyes closed conditions, participants were instructed to first gaze at the designated target with their eyes open and then to close their eyes while imagining that they are still gazing at the target. This instruction was specifically provided to match the eyes-in-head position between the eyes-open (EO) and the eyes-closed (EC) conditions. Five repetitions (13) of each condition were performed in a random order using a Latin rectangle. Rest between trials was provided as necessary without any restrictions. Participants that reported using corrective visual aids for usual daily activities (myopia but not presbyopia) were tested with their own aids. To ensure compliance with the instructions, one of the investigators stood by the participants throughout the experiment and watched their gaze behavior (when possible).

### 2.3 Instruments

To measure sway during the trials, participants stood on a platform equipped with an embedded force sensor array (Zebris FDM-T Treadmill, *Zebris Medical GmbH*, Germany). For consistency, the standardized foot position was marked on the platform. Raw data were acquired using the software provided by the manufacturer (Zebris FDM, version 1.18.40) at a 60 Hz sampling rate. Since the platform is elevated from the laboratory’s floor, a 1.70 × 0.7 × 0.3 m wooden platform was custom built to create an illusion of continuity of the standing surface (on which the target for the downward gaze was placed), and participants were tested while facing the back end of the treadmill.

To measure head angle throughout the experiment, participants were fitted with a single inertial measuring unit (IMU) on their forehead. The IMU (Xsens DOT, Movella, Netherlands) was placed in a special case attached to an elastic band, designed for this purpose (provided by the manufacturer). Data from the IMU was sampled at 60 Hz and acquired wirelessly using software provided by the manufacturer (Version 2020.0.1).

### 2.4 Data processing and outcome measures

Raw data from the force platform was exported and processed by a dedicated MATLAB script. First, the center of pressure (COP) time series was low passed using a 2nd order Butterworth filter with a cutoff frequency of 15 Hz. The script, which excludes the first 3 s and the last second of each trial, computes four traditional sway parameters from the individual time series: COP range in the anterior–posterior (AP) and medio-lateral (ML) directions is simply the distance between the extreme values on the Y and X axes, respectively, and given in mm. Sway velocity, given in mm/s, was calculated as the total excursion divided by time. The fourth parameter was sway area (given in mm^2^). We chose to calculate the area of the smallest convex set containing all visited points (convex hull) of the 2D data [as described in Wollseifen (14)].

As the main outcome measure, the script computes the short-term diffusion coefficient of COP, driven from stabilogram diffusion analysis (SDA) as described by Collins and De-Luca (15). Briefly, the diffusion coefficient is the rate at which the quadratic Euclidean distance between two COP positions increases as a function of the time interval between them. That is, for a given Δt, spanning m data intervals and N samples, planar displacement (Δr^2^) is calculated as:


$$\mathrm{Drs}=\langle \Delta r^{2}\rangle =\frac{\sum_{i=1}^{N-m\;}{\left({\mathrm{\Delta r}}_{i}\right)}^{2}}{\left(N-m\right)}$$

This calculation is repeated for every Δt, and the Dis is calculated as the slope of the Δi^2^ by Δt plot. In this experiment we calculated three coefficients: single dimension on the X- (Dxs) and Y- (Dys) axes, and the planar coefficient (Drs), all given in mm^2^/s.

SDA parameters were shown to be more sensitive than summary statistics (traditional parameters) to postural instability (12). Nevertheless, many researchers prefer using traditional sway metrics, so we included both. Regardless of the true nature of standing sway (which is debated in the literature), the task in this study required participants to minimize sway as much as possible; therefore, smaller values (of all parameters) are interpreted as a better ability to control the COM.

To determine the vertical head angle in each trial, the data from the force platform and the vertical angle (relative to the gravitational vector) data from the IMU were synchronized using the time stamps from the devices. The head angle was determined as the mean value during the trial.

### 2.5 Sample size estimate

To estimate the number of participants required to show an effect of DWG on postural sway, we used the data collected from our previous study, which included four participants (one older adult and three PwS) who were tested in a wide-base stance (9). We used the ‘SIMR’ package (16) in R (Version 4.0.5), in conjunction with the ‘lme4’ package (17). This package allows users to calculate power for generalized linear mixed models. The power calculations are based on Monte Carlo simulations (16). We simulated multiple experiments with DWG (to 3 meters ahead) and forward gazing (FG) as levels of the fixed effect, at various levels of the random effect (i.e., number of participants). When the predicted term in these simulations was the variable Drs, the observed power reached 92% (CI: 85–96) with 20 participants. Given that we were (mostly) interested in the interaction term (‘Vision’ by ‘Angle’) and not the main effect of the angle, we decided to recruit 20 participants in each group. This value is much greater than that previously estimated (9), and is probably the result of testing participants in a wide-base-stance instead of narrow-base-stance, which is more sensitive to postural instability (18).

### 2.6 Statistical analysis

In all cases, mixed-effects models were used for the analysis (using SPSS, Version 29, IBM Corp, Armonk, NY). For the main objective, models included participants as the random effect. For fixed effects, we used ‘Group’, Vision’, ‘Angle’, and all possible interactions (full factorial models). Non-significant terms were excluded from the model in a stepwise manner. Significance level was set a-priori at *α* < 0.05, and sequential Bonferroni was used to correct for multiple comparisons when appropriate. For effect size, we used the marginal pseudo *R*^2^, which quantifies the variance explained by the fixed effects in the model. The distribution of all sway parameters was skewed to the right and therefore adjusted using a logarithmic transformation (natural log). The residuals of all models were evaluated for their distribution.

## 3 Results

One PwS did not complete the full experimental protocol due to tiredness. This participant performed only four trials of each of the conditions. Data from six other trials (from three different participants) were excluded from the final analysis due to interruptions (participant either moved or talked, or other disturbance occurred during the trial). Overall, sway data from 790/796 trials exectuted, performed by 20 healthy adults and 20 PwS, were used in the final analysis. Participants’ characteristics are presented in Table 1.

**Table 1:** Participants’ characteristics.

Characteristic	Healthy		Stroke	
	Men	Women	Men	Women
*N*	10	10	15	5
Height cm	176 [165–185]	161 [153–170]	171 [156–184]	159 [154–167]
Weight kg	85 [71–121]	65 [52–89]	80 [59–105]	63 [48–79]
BMI kg/m^2^	27.3 [23.3–35.3]	25.3 [19.8–35.8]	27.3 [20.1–32.6]	24.9 [20.2–31.2]
Age years	49 [26–75]	44 [25–68]	61 [30–76]	71 [64–77]
TFO months			3 [1–26]	
FM-LE			23.7 [6–34]	
10MWT m/s			0.8 [0–1.4]	
Affected side R/L			13/7	
Stroke type I/H			14/6	

^*^Values represent mean and [range]. BMI, body-mass-index, TFO is the time (in months) from stroke onset. FM-LE is the motor section of the Fugl-Meyer lower extremity (max score is 34), and the 10MWT is the score (mean walking velocity) in the 10-meter walk test (with 0 meaning that the participant was unable to perform the task). R-right, L-left, I-ischemic and H-hemorrhagic.

### 3.1 Head angle

First, we wanted to ensure that head angles in the EO and EC conditions were similar, and to estimate whether participants lowered their heads (head-on-neck) or eyes (eyes-in-head) to gaze down. The results of this comparison revealed that the DWG head angle differed from the FG head angle by roughly 20° (*p* < 0.0001), indicating that DWG was achieved (at least partially) using head-on-neck movement. The mean difference between the EO and EC conditions was <0.5° (DWG = 0.57° and FG = 0.12°) and was not significant (*p* > 0.39).

### 3.2 Main results

**Figure 1:**
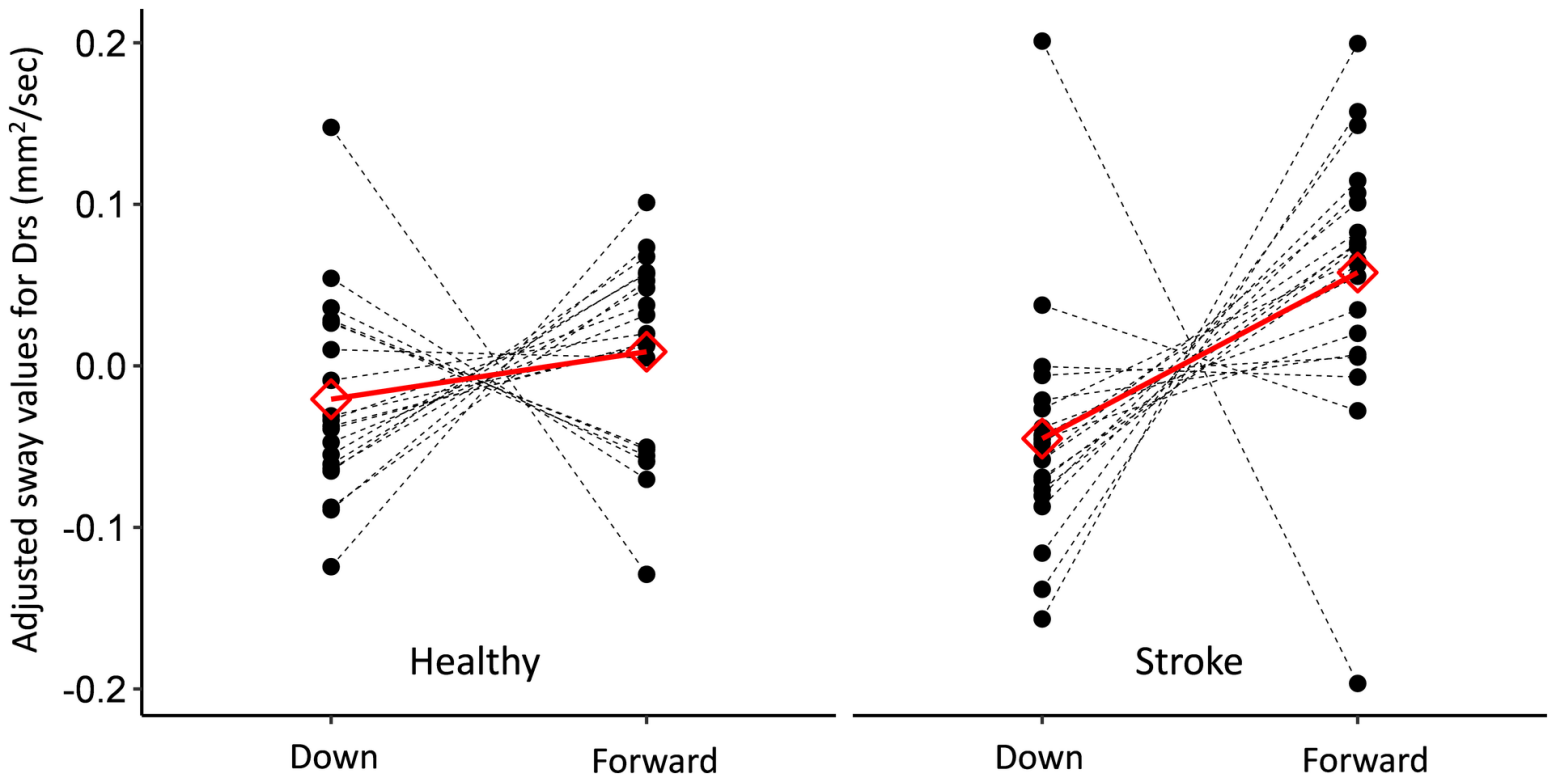
Between-group comparison of the participants’ individual (black) and the mean (red) responses to the gaze angle. Results are presented as a within-subject effect (after adjusting for the random intercept), for the parameter Drs. While the response in the stroke group was greater (in terms of magnitude and the number of participants responding in the direction of the mean response) than that observed in the control (healthy) group, this difference was not significant for the parameter Drs; however, it was significant in other models.

**Figure 2:**
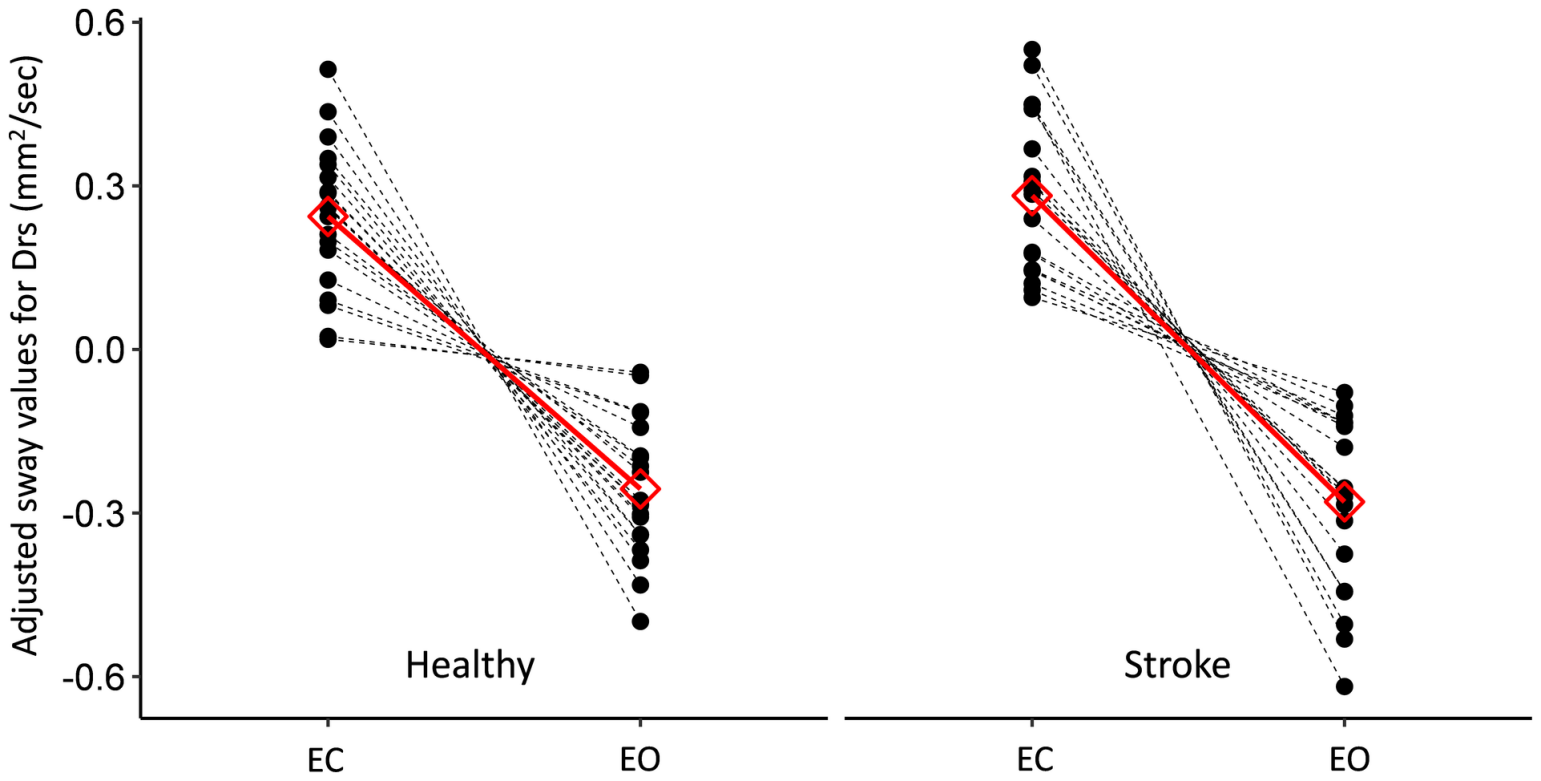
Between-group comparison of the participants’ individual (black) and the mean (red) responses to the visual condition. Results are presented as a within-subject effect (after adjusting for the random intercept) for the parameter Drs. EC and EO stand for eyes closed and eyes open, respectively.

The effects of the ‘Group’, ‘Vision’, ‘Angle’ and the ‘Group’-by-‘Angle’ interaction, on multiple sway metrics (see Methods), were tested. The results of these tests are presented in Table 2 and in Figures 1, 2.

**Table 2:** Final models for all sway outcome measures.

Parameter	Group	Vision	Angle	G × A	*R* ^2^
Drs	1.13 [0.62–1.64] *p* = 1.5 × 10^−5^	0.53 [0.44–0.61] *p* < 0.001	0.065 [0.021–0.11] *p* = 0.004		0.32
Dys	0.99 [0.51–1.47] *p* = 5.1 × 10^−5^	0.60 [0.51–0.69] *p* < 0.001	0.064 [0.011–0.12] *p* = 0.02		0.30
Dxs	1.17 [0.54–1.178] *p* < 0.001	0.35 [0.26–0.43] *p* = 2.2 × 10^−14^	0.066 [0.011–0.12] *p* = 0.02	*p* = 0.02	0.23
RangeX	0.53 [0.26–0.80] *p* < 0.001	0.094 [0.05–0.14] *p* = 5 × 10^−5^	0.001 [−0.030–0.033] *p* = 0.94	*p* = 0.001	0.21
RangeY	0.41 [0.18–0.63] *p* < 0.001	0.17 [0.13–0.26] *p* = 5.1 × 10^−13^	0.024 [−008–0.057] *p* = 0.14	*p* = 0.02	0.20
Area	0.93 [0.48–1.39] *p* = 6.9 × 10^−5^	0.27 [0.20–0.34] *p* = 5.1 × 10^−14^	0.029 [−0.023–0.082] *p* = 0.28	*p* = 0.003	0.25
Velocity	0.53 [0.28–0.78] *p* = 3 × 10^−5^	0.28 [0.24–0.33] *p* < 0.001	0.043 [0.023–0.063] *p* = 3.9 × 10^−5^	*p* = 0.04	0.32

Values are the mean difference [95% CI] between the two levels of each main (fixed) effect. For the Group by Angle (G × A) interaction, only the p-value is reported. *R*^2^ is the marginal pseudo *R*^2^, which quantifies the variance explained solely by the fixed effects in the model.

Briefly, for all outcome measures we found the ‘Group’ and ‘Vision’ terms to be significant, revealing that mean sway values in the stroke group and with eyes closed, were significantly greater than the mean values in the healthy group and with eyes open, respectively.

The ‘Angle’ term was found to be significant in four out of the seven models, suggesting that mean sway values in the FG condition were significantly greater than the mean values in the DWG condition. Nevertheless, a significant ‘Group’ by ‘Angle’ interaction (5/7 models) revealed that FG was indeed associated with increased sway values in all models, but that this effect was either observed only in the stroke group or was more pronounced in the stroke group. None of the other interactions were found to be significant. Importantly, none of the statistical models revealed a significant ‘Vision’-by-‘Angle’ interaction, as would be predicted from our hypothesis.

## 4 Discussion

The main objective of this study was to provide evidence to support the hypothesis that the greater postural steadiness observed when a person gazes down is primarily a result of altered visual input. The results of this study revealed that three factors had a negative influence on the ability of participants to attenuate their body sway (i.e., to control posture): lack of visual input, a previous stroke, and a straight (horizontal) gaze angle. Importantly, our results indicate that retinal input had played no role in the effect of DWG on postural control, and PwS seem to be more sensitive than controls to the effect of DWG. The observed effects of visual input and of stroke are consistent with current literature, including previous reports from our own laboratory (8, 9) and will not be further discussed.

DWG is a common clinical observation among PwS (4) and other unstable walkers. While this behavior is often assumed to support online stepping control, there are other possibilities, including postural control (8, 9) and a way to free cognitive resources i.e., disengaging from distracting visual input (19). In all these cases, it would be reasonable to assume that DWG is used to change, in some way, the visual input. Nevertheless, most previous investigations in this area controlled for the visual input (5, 6, 20, 21), eliminating its effect. This approach is quite reasonable given that the visual input depends on the visual structure of the environment [e.g., Simoneau et al. (20)], which has endless possibilities. Yet, this approach does not allow to determine how DWG behavior affects postural control during daily life. Further, in daily life, DWG is achieved by a combination of downward head and eye movements (22), which seem to have opposite effects on postural control [see the difference between (5, 6)].

To investigate whether visual input plays a role in the effect of DWG on postural control during daily life, we simply instructed participants to gaze down (allowing them to choose whether to use head-on-neck, eyes-in-head or both, to do so). We then tested whether visual input modulates the effect of DWG, as indicated by the interaction term in the statistical models. While several other authors (4, 5, 7, 23) have used a similar approach, none directly tested the interaction term. Instead, they reported, for each visual condition, whether sway values were significantly different between gaze angles. While this approach is often acceptable, it does not necessarily indicate whether the magnitude of the effect changes (24). The main results of this investigation do not point to any such modulatory effect of the visual input, indicating that the observed effect is related to a different sensory modality (or modalities), or to a biomechanical effect (5). However, that we found no modulatory effect of visual input does not mean it plays no role in DWG’s impact on postural sway. Rather, it suggests DWG can enhance postural control even without visual input. Nonetheless, the current results do provide important information: first, the main effect of the gaze angle replicates our previous findings (8, 9) even though we used different settings, including measuring device, environment, gaze distance, stance width and a different sample, thus providing evidence that our initial findings were not coincidental. These results are consistent with reports on the stabilizing effect of downward eye movement (2, 6), in which the visual structure of the environment was controlled (to some extent). Interestingly, this effect seems to be more prominent at far distances (2), and suggested to result from afferent/efferent signals of the extraocular, and/or neck muscles. However, these results are inconsistent with a report (5) on the destabilizing effect of downward head motion (in which the visual structure of the environment was controlled by both matching it and by eliminating it). These authors suggested a biomechanical explanation, but had used a much larger (twice as large) head angle than that observed in the current report.

Second, in the current investigation we found a significant Group by Angle interaction, indicating that PwS are more sensitive to the effect of DWG, a finding consistent with a previous report (4) but inconsistent with our own previous investigation (9). We believe that two factors contributed to this result: stance width and sample size. Specifically, in our previous report participants were tested in a narrow-base stance and in the current in a wide-base stance. Narrow-base stance was reported to increase the sensitivity of sway measurements to instability (18), possibly, due to the greater postural challenge. This possibility is consistent with a previous report (7) showing that DWG enhanced postural steadiness of healthy adults standing on one leg, an effect that disappeared when participants were tested in a two-legged stance.

Another (or maybe additional) possibility, is that our previous report was underpowered to detect such interaction. In our previous report, only 10 PwS were tested because we were interested only in the main effect of gaze angle. In our current report, 20 PwS were tested, because we were more interested in the interaction terms, which require a greater sample. Also noteworthy is the fact that the interaction term was found significant in models for traditional sway metrics, which we did not use in the aforementioned investigation. These facts, among others, can also explain the differences between our results and those reported by Aoki et al. (4).

Third, as opposed to the common assumption that DWG is about changing/manipulating visual input, the results presented here suggest otherwise. Hayhoe and Matthis (3) state that gaze behavior investigations are challenging, because it is impossible to know what visual information is being acquired and for what purpose. We wish to extend this notion by adding that the results of the current investigation imply that “gaze behavior “is not necessarily about visual input altogether. Instead, the downward position of the head and eyes can serve to enhance some other sensory signal or enhance postural control through some other mechanism (for example, altered biomechanics), which is unknown at this time. From a clinical perspective this distinction might be important. Specifically, a patient may gaze down for online control over stepping, possibly because he/she does not trust their ability to react to slips and trips. For such patients, reactive training could be beneficial [see for example (25)], while for those gazing down for postural control more traditional balance training could be beneficial. For these individuals, we would suggest training without visual input to begin with, both because the DWG is likely to indicate deficit in some other feedback loop, and because training without visual input can prevent compensation through the visual system.

Naturally, this study has several limitations, two of which are addressed below. The first limitation is the fact that we talk about “gaze” but only measure head angle without the concurrent eyes angle. This fact introduces two problems: (1) we are unable to quantitatively assess the compliance of participants with our instructions, and to test whether the downward eyes angle was matched between the EO and EC conditions. (2) We are unable to determine whether downward eyes-in-head movement even occurred. The second is easily resolved by calculating the required gaze angle to look 2 m ahead, using a simple geometrical model. For example, if we consider the shortest participant in the study (153 cm), who requires the smallest DWG angle. For this participant, roughly 38° of downward gaze angle is required to look down 2 m ahead. Our data indicates that this participant used a ~ 15° downward head angle, meaning that the difference was achieved by downward eyes movement. As for the first problem, while some qualitative assessment of compliance was used (see Methods), this is indeed a limitation that should be considered when interpreting the results.

The second limitation is the fact that we did not age-matched our participants, which makes it impossible to conclude whether the difference between groups is a result of their stroke, their age or both. While true, we found no age effect in any of the groups (see Supplementary Figure S1, Supplemental Digital Content 1, for graphic presentation of the relation between age and sway values). In fact, when dividing the groups to younger (≤60) and older (>60), no difference was observed between them (also presented graphically in Supplementary Figure S1). This is likely due to some sampling bias of the older adults, as the effect of aging is well known, including our own observations (9). Nevertheless, to test whether stroke or instability as general is what made the stroke group more sensitive to the effect of DWG, future investigations should concentrate on other unstable groups, while appropriately controlling for age.

In conclusion, the results of the current report indicate that DWG can enhance postural control (replicating previous reports from our laboratory) even in the absence of visual input. These results also suggest that the effect of DWG is more pronounced in PwS, possibly due to their general instability. It is important to keep in mind that DWG prevent the walker from acquiring and using visual information about the far environment, information that is useful for navigation, planning a future trajectory and anticipating future disturbances, which can lead to reduced walking velocity, increase the risk of falls and, over time, can cause a shift from healthy automaticity to compensatory conscious control (26), even in those that gaze down for postural control.

## Data Availability

The raw data supporting the conclusions of this article will be made available by the authors, without undue reservation.

## Author’s note

An early version of this manuscript was made available as a preprint, and can be found in https://doi.org/10.1101/2023.12.20.23300291.

## Supplementary material

SUPPLEMENTARY FIGURE S1 The effect of Age on postural sway. Given that participants in the stroke group were older than those in the control group, we explored the effect of age on postural sway. To do so, we plotted the mean sway value (of each participant) by age (left panel). Visually evaluating this plot revealed no obvious relation between the two variables. In addition (on the right), we classified participants as ‘young’ (<60 years) and ‘old’ (>60 years) and compared sway values between classes within each group. Comparison revealed no difference between age classes (*p* > 0.6). The mean and 95%CI of each group and age class are presented. Both panels show no obvious effect for the age of participants.

## References

[1] Peterka RJ. Sensorimotor integration in human postural control. J Neurophysiol. (2002) 88:1097–118. doi: 10.1152/jn.2002.88.3.1097, PMID: 12205132

[2] Kapoula Z Le TT. Effects of distance and gaze position on postural stability in young and old subjects. Exp Brain Res. (2006) 173:438–45. doi: 10.1007/s00221-006-0382-1, PMID: 16525804

[3] Hayhoe MM Matthis JS. Control of gaze in natural environments: effects of rewards and costs, uncertainty and memory in target selection. Interface Focus. (2018) 8:20180009. doi: 10.1098/rsfs.2018.0009, PMID: 29951189 PMC6015808

[4] Aoki O Otani Y Morishita S Domen K. Influence of gaze distance and downward gazing on postural sway in hemiplegic stroke patients. Exp Brain Res. (2014) 232:535–43. doi: 10.1007/s00221-013-3762-3, PMID: 24253441

[5] Buckley JG Anand V Scally A Elliott DB. Does head extension and flexion increase postural instability in elderly subjects when visual information is kept constant? Gait Posture. (2005) 21:59–64. doi: 10.1016/j.gaitpost.2003.11.005, PMID: 15536034

[6] Ustinova K Perkins J. Gaze and viewing angle influence visual stabilization of upright posture. Brain Behav. (2011) 1:19–25. doi: 10.1002/brb3.10, PMID: 22398978 PMC3217671

[7] Aoki O Otani Y Morishita S Domen K. Effects of viewing distance and head flexion on postural control during one and two-legged stance. Int J Physiother Res. (2015) 3:1215–20. doi: 10.16965/ijpr.2015.179

[8] Koren Y Mairon R Sofer I Parmet Y Ben-Shahar O Bar-Haim S. Gazing down increases standing and walking postural steadiness. R Soc Open Sci. (2021) 8:201556. doi: 10.1098/rsos.201556, PMID: 33959324 PMC8074885

[9] Koren Y Handelzalts S Parmet Y Bar-Haim S. Older adults and stroke survivors are steadier when gazing down. PLoS One. (2023) 18:e0285361. doi: 10.1371/journal.pone.0285361, PMID: 37205706 PMC10198484

[10] Franz JR Francis CA Allen MS O’Connor SM Thelen DG. Advanced age brings a greater reliance on visual feedback to maintain balance during walking. Hum Mov Sci. (2015) 40:381–92. doi: 10.1016/j.humov.2015.01.012, PMID: 25687664 PMC4372858

[11] Bonan IV Colle FM Guichard JP Vicaut E Eisenfisz M Tran Ba Huy P. Reliance on visual information after stroke. Part I: balance on dynamic posturography. Arch Phys Med Rehabil. (2004) 85:268–73. doi: 10.1016/j.apmr.2003.06.017, PMID: 14966712

[12] Collins JJ De Luca CJ Burrows A Lipsitz LA. Age-related changes in open-loop and closed-loop postural control mechanisms. Exp Brain Res. (1995) 104:480–92. doi: 10.1007/bf00231982, PMID: 7589299

[13] Meyer P. The role of plantar cutaneous afferents in quasi-static and dynamic balance control In: Chapter 3: Reliability of stabilogram–diffusion parameters for postural analysis (2003)

[14] Wollseifen T. Different methods of calculating body sway area. Pharm Programm. (2011) 4:91–106. doi: 10.1179/175709311X13166801334271

[15] Collins JJ De Luca CJ. Open-loop and closed-loop control of posture: a random-walk analysis of center-of-pressure trajectories. Exp Brain Res. (1993) 95:308–18. doi: 10.1007/BF00229788, PMID: 8224055

[16] Green P MacLeod CJ. SIMR: an R package for power analysis of generalized linear mixed models by simulation. Methods Ecol Evol. (2016) 7:493–8. doi: 10.1111/2041-210X.12504

[17] Bates D Mächler M Bolker B Walker S. Fitting linear mixed-effects models using lme4. J. Stat. Softw. 67, 1–48. (2015).

[18] Melzer I Benjuya N Kaplanski J. Postural stability in the elderly: a comparison between fallers and non-fallers. Age Ageing. (2004) 33:602–7. doi: 10.1093/ageing/afh218, PMID: 15501837

[19] Ellmers TJ Cocks AJ Doumas M Williams AM Young WR. Gazing into thin air: the dual-task costs of movement planning and execution during adaptive gait. PLoS One. (2016) 11:e0166063. doi: 10.1371/journal.pone.0166063, PMID: 27824937 PMC5100909

[20] Simoneau GG Leibowitz HW Ulbrecht JS Tyrrell RA Cavanagh PR. The effects of visual factors and head orientation on postural steadiness in women 55 to 70 years of age. J Gerontol. (1992) 47:M151–8. doi: 10.1093/geronj/47.5.m151, PMID: 1512430

[21] Vuillerme N Rougier P. Effects of head extension on undisturbed upright stance control in humans. Gait Posture. (2005) 21:318–25. doi: 10.1016/j.gaitpost.2004.04.007, PMID: 15760748

[22] Thomas NDA Gardiner JD Crompton RH Lawson R. Look out: an exploratory study assessing how gaze (eye angle and head angle) and gait speed are influenced by surface complexity. Peer J. (2020) 8:e8838. doi: 10.7717/peerj.8838, PMID: 32280566 PMC7134013

[23] Aoki O Otani Y Morishita S Domen K. The effects of various visual conditions on trunk control during ambulation in chronic post stroke patients. Gait Posture. (2017) 52:301–7. doi: 10.1016/j.gaitpost.2016.12.018, PMID: 28033576

[24] Makin TR Orban de Xivry J. Ten common statistical mistakes to watch out for when writing or reviewing a manuscript. eLife. (2019) 8:e48175. doi: 10.7554/eLife.48175, PMID: 31596231 PMC6785265

[25] Batcir S Shani G Shapiro A Melzer I. Characteristics of step responses following varying magnitudes of unexpected lateral perturbations during standing among older people - a cross-sectional laboratory-based study. BMC Geriatr. (2022) 22:400. doi: 10.1186/s12877-022-03080-w, PMID: 35524172 PMC9078012

[26] Clark DJ. Automaticity of walking: functional significance, mechanisms, measurement and rehabilitation strategies. Front Hum Neurosci. (2015) 9:246. doi: 10.3389/fnhum.2015.00246 25999838 PMC4419715

