# Supplementary figures and images for: Downward gazing behavior after stroke can enhance postural control even in the absence of visual input
Source: Front Neurol. 2025 May 14;16:1593221. doi: 10.3389/fneur.2025.1593221 (PMC12117824; doi:10.3389/fneur.2025.1593221)

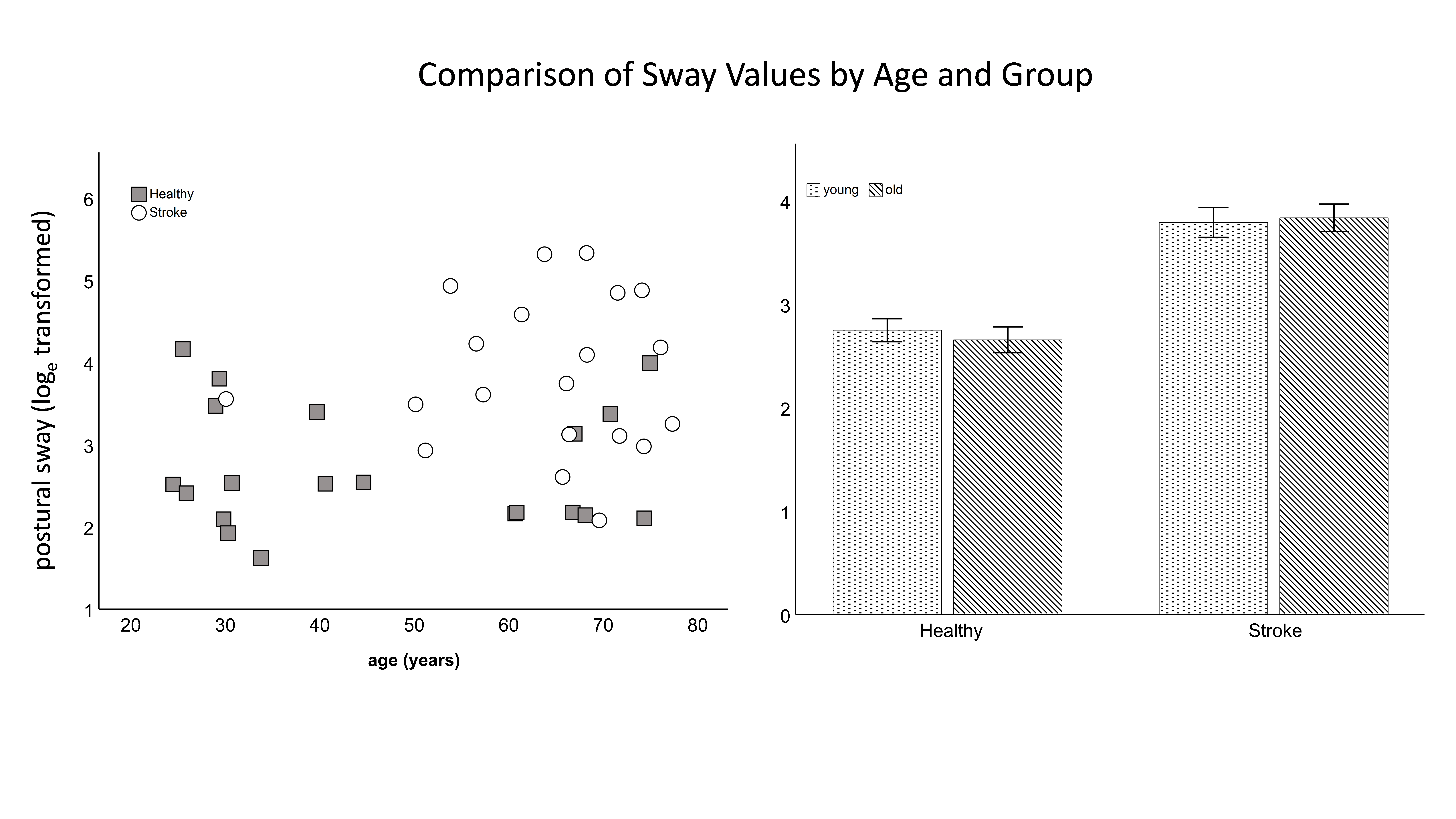

Supplement: SUPPLEMENTARY FIGURE S1 — The effect of Age on postural sway. Given that participants in the stroke group were older than those in the control group, we explored the effect of age on postural sway. To do so, we plotted the mean sway value (of each participant) by age (left panel). Visually evaluating this plot revealed no obvious relation between the two variables. In addition (on the right), we classified participants as ‘young’ (<60 years) and ‘old’ (>60 years) and compared sway values between classes within each group. Comparison revealed no difference between age classes (p > 0.6). The mean and 95%CI of each group and age class are presented. Both panels show no obvious effect for the age of participants. [file Image_1.TIF]
